# Rethinking cardiac neurodevelopment: a dimensional model of adversity in congenital heart disease

**DOI:** 10.3389/fped.2026.1888600

**Published:** 2026-07-31

**Authors:** Adam R. Cassidy

**Affiliations:** Departments of Psychiatry & Psychology and Pediatric & Adolescent Medicine, Mayo Clinic, Rochester, MN, United States

**Keywords:** adversity, brain development, congenital heart disease - cardiac, Dimensional Model of Adversity and Psychopathology (DMAP), neurodevelopment

## Abstract

Despite major advances in cardiac care and decades of research, neurodevelopmental (ND) outcomes among children with congenital heart disease (CHD) have shown little improvement over time, substantial variability in outcomes remains unexplained, and prediction of individual developmental trajectories remains limited. This article argues that one contributor to these limitations is the field's reliance on descriptive but mechanistically underspecified models that catalog risk factors without clearly explaining how specific environmental experiences influence neurobehavioral development. To address this gap, this paper proposes an integrated dimensional model of neurodevelopment in CHD that conceptualizes ND outcomes as arising from the independent and interactive effects of CHD-related brain vulnerability and exposure to environmental medical adversity organized along the dimensions of deprivation and threat. Drawing from the Dimensional Model of Adversity and Psychopathology, the framework integrates prenatal dysmaturation, perioperative brain injury, and persistent alterations in brain structure and connectivity with adverse experiences occurring across prenatal, intensive care, caregiving, educational, and socioeconomic contexts. The model predicts that deprivation-related exposures will preferentially affect cognitive, language, and executive functioning systems, whereas threat-related exposures will more strongly influence emotional learning, stress regulation, and risk for psychopathology. By organizing CHD-related experiences according to developmentally meaningful dimensions of adversity, this framework provides a more mechanistic account of ND heterogeneity in CHD, generates testable hypotheses regarding developmental trajectories, and may support more precise developmental surveillance and targeted intervention strategies.

## Introduction

As medical advances have led to longer, healthier, and more productive lives for children born with congenital heart disease (CHD), attention has shifted to the neurodevelopmental (ND) challenges these individuals face. For many, ND difficulties are among the most consequential and persistent complications of CHD, often undermining patient and family quality of life to an even greater degree than medical or surgical morbidities (1). Recognition of the importance of ND monitoring and intervention has grown substantially over recent years, driven by several impactful scientific statements from the American Heart Association (1–3), the formation of multidisciplinary organizations dedicated to optimizing ND outcomes among individuals with CHD [e.g., the Cardiac Neurodevelopmental Outcome Collaborative, CNOC (4)], and the ongoing efforts of clinicians, researchers, scientists, and stakeholders worldwide.

Advancements in cardiac ND research have been substantial, particularly given the field's relatively recent establishment. However, as we consider future directions and where the field should go from here, it is essential to address three significant unresolved challenges:

1. ND outcomes among children with CHD have remained stubbornly unchanged over time, with no appreciable signs of meaningful improvement despite advances in detection, diagnosis, and medical/surgical management (5, 6).
2. Despite decades of research aimed at identifying risk factors (e.g., genetic, birth, medical, surgical, perioperative, socioeconomic) for poor ND outcomes among children with CHD, our best models still explain only about 30% of the variability in those outcomes (1, 5).
3. Even as we come to more fully appreciate the many varied ways CHD can undermine cognitive, behavioral, emotional, and social development (7), our capacity to predict *which* children will face ND difficulties – and the *specific nature of those difficulties* – remains limited (8).

These problems are not insignificant, nor are they insurmountable, provided we have an accurate understanding of their root causes and contributing factors. One such factor, I contend, is our adherence to descriptive but mechanistically underspecified models of cardiac neurodevelopment that focus on cataloging environmental risks without fully articulating *why* various exposures are relevant or *how* they impact *specific* ND systems. A more mechanistic developmental framework is needed.

I propose an integrated model wherein ND outcomes in children with CHD are shaped by the individual and interactive effects of biological/brain vulnerability associated with CHD and exposure to environmental medical adversity, conceptualized across the dimensions of deprivation and threat.

To support this proposal, I will begin with a brief historical overview of cardiac ND research, focusing specifically on the implicit model that the field has been using to date, the latent organizing assumptions of that model, and why those assumptions are limiting. Next, I will describe a dimensional model of adversity and its utility for understanding and predicting development and psychopathology. I will then outline the main elements of an integrated cardiac ND model, including evidence of neurological vulnerabilities linked to complex CHD and how the diverse environmental experiences of children with CHD can be seen through the framework of dimensional adversity. Finally, I will synthesize these key brain and environmental components into a comprehensive cardiac ND model, proffer several testable hypotheses that arise from this model, and address some potential challenges and important future directions.

## A brief historical overview and critique of the current model of cardiac ND research

Research into the ND sequelae of CHD began in earnest during the 1980s, in response to dramatic medical and surgical advances that started to allow children with complex CHD to survive and live longer than ever before. Initially, the emphasis of this research was on biology and the search for modifiable intraoperative factors that could be optimized to reduce the risk for brain injury (9). As these children got older and more able to complete comprehensive neuropsychological assessment, efforts expanded to characterize short- and longer-range outcomes in CHD and chart the many varied ways children and adolescents with complex CHD may differ from their typically developing peers (10–16). From there, researchers turned their attention to prediction; to identifying those children with CHD who may be at particularly high ND risk. The advent of sophisticated fetal neuroimaging (17–20) and high-throughput genetic analysis (21, 22) permitted ever greater insights into the origins of ND impairment in CHD; and increasing appreciation of the social drivers of health (23–25) shone a powerful spotlight on sociodemographic and socioeconomic influences, which often account for more variance in ND outcomes than medical/surgical factors (5). However, even these essential improvements did not fundamentally reorganize the field's conceptual model.

There is now widespread recognition that ND outcomes in CHD are complex and multidetermined, influenced by biological, genetic, and environmental factors (1). Yet, substantial heterogeneity remains insufficiently explained. Across cardiac ND studies, an implicit framework exists in which 1) outcomes are often framed as the direct downstream effects of biology and/or brain injury, 2) risk factors are grouped by tradition/convenience (e.g., “birth,” “medical/surgical,” “sociodemographic”) rather than developmental relevance, 3) adversity within the context of CHD is undifferentiated, often operationalized via multifactorial proxy variables (e.g., length of hospital stay, socioeconomic status), and 4) variability in ND outcomes is described but not mechanistically explained. Consequently, despite years of study, we are still unable to explain why children with similar cardiac anatomy can have dramatically different outcomes. Our coarse conceptualization of CHD-related environmental exposures does not map on to specific neurobehavioral systems. And without a clearer picture of which potentially modifiable environmental risk factors exert the greatest developmental impact, our ability to implement effective interventions remains limited.

What is lacking is not recognition that environment matters, but rather a framework for conceptualizing CHD-related environmental experiences that is mechanistically informative and developmentally meaningful (i.e., ecologically valid). A dimensional approach offers one such framework.

## Dimensional models of adversity in developmental science

Developmental science has long sought to understand which early experiences shape development, why those experiences matter, and how they influence specific neurobehavioral outcomes (26). One particularly influential framework is the *Dimensional Model of Adversity and Psychopathology (DMAP)*, proposed by McLaughlin and Sheridan (27, 28).

Unlike cumulative risk models, which focus on the quantity of adverse experiences (29), the DMAP distinguishes between qualitatively distinct dimensions of adversity (27). Two primary dimensions have been identified: *deprivation*, defined as the absence of developmentally appropriate environmental stimulation, and *threat*, defined as exposure to harm or the threat of harm (27, 28, 30). Grounded in neuroscientific theories of experience-expectant and experience-dependent brain development (31), the DMAP posits that these dimensions influence distinct neural systems and learning processes.

Deprivation is thought to affect cognitive development by limiting the environmental stimulation necessary for the formation and refinement of neural networks. When children lack opportunities for rich cognitive, linguistic, and social interaction, neural circuits supporting higher-order cognition – particularly within association cortices of the prefrontal, parietal, and temporal lobes – may develop less efficiently (28, 32–35). As a result, deprivation has been most consistently linked with deficits in executive function, language, and academic learning (26–28, 34).

In contrast, threat influences development primarily through its impact on stress-response systems and emotional learning circuitry, including the amygdala, hippocampus, and ventromedial prefrontal cortex (26). Exposure to threatening environments can heighten sensitivity to potential danger and alter fear-learning processes. Empirical studies have shown that children exposed to maltreatment or violence exhibit heightened attention to threat-related cues and alterations in fear conditioning, which are associated with increased risk for both internalizing and externalizing psychopathology (36–39). Although exposure to threat is often linked with poorer cognition as well, these relationships are frequently confounded by concurrent experiences of deprivation. Research that separates these factors indicates that deprivation is a more robust predictor of cognitive outcomes, whereas threat is more closely tied to emotional and behavioral dysregulation (32, 35, 40).

Historically, studies looking at ND outcomes in cardiac populations have focused on complex risk factors such as length of hospital stay (LOS), which has been found to be an important predictor of long-term outcomes (1, 41, 42). However, LOS does not readily facilitate a clear or mechanistic understanding of how, and in what ways, extended hospitalization adversely affects child neurodevelopment. It remains unclear whether LOS serves merely as an indicator of the underlying complexity of the cardiac condition, or if its impact arises from associated factors like increased frequency of painful procedures, sedation, restricted mobility and exploration, or separation from caregivers. In reality, LOS likely represents a complex combination of deprivation and threat exposures that vary across patients. Without more systematic efforts to disentangle the specific exposures that occur within the intensive care unit and their distinct effects on neurobehavioral systems, our understanding of why certain children experience better or worse outcomes remains limited. This knowledge gap hampers the development of targeted interventions designed to address the most significant risks these children face.

## Key elements of an integrated dimensional model of cardiac ND

Dimensional frameworks such as the DMAP offer valuable perspectives for examining the impact of adversity on individual ND trajectories, particularly when integrated with relevant biological characteristics. In predicting ND outcomes among individuals with CHD, it is essential to consider both the intrinsic brain vulnerabilities linked to complex cardiac conditions and how those vulnerabilities may interact with the environments to which they are exposed.

### Brain vulnerability in CHD

Complex CHD can profoundly impact brain development. A comprehensive review of the effects of CHD on brain development is beyond the scope of this paper; interested readers are encouraged to see prior reviews [e.g. (1, 43–45),].

Briefly, brain development in complex CHD begins to diverge from expected trajectories *in utero*, during the third prenatal trimester (17, 18). These differences are thought to arise primarily from alterations in fetal circulation (46, 47), placental insufficiency (48–50), and genetic influences (21, 51). As a result, full-term newborns with complex CHD often exhibit patterns of brain maturation resembling those of fetuses at 34–36-weeks gestation (52).

Even before undergoing cardiac surgery, infants with complex CHD exhibit microstructural dysmaturation of the cerebral cortex stemming from impaired dendritic arborization (53) and high rates of brain injury. Preoperative brain injury has been reported in up to 34% of infants with dextro-transposition of the great arteries and 49% of infants with left-sided heart lesions (54). White matter injury is particularly common in CHD, occurring prior to surgery in approximately 20% of infants undergoing cardiac surgery before 8 weeks of age, and an additional 44% of infants following surgery (55).

Importantly, these early differences in brain macro- and microstructure do not appear to resolve over time. Neuroimaging studies have documented persistent differences in brain structure and connectivity across childhood, adolescence, and adulthood (10–12, 56, 57), with one systematic review reporting a nearly 8-fold greater risk of having brain abnormalities among adolescents/young adults with CHD (58).

Taken together, these findings suggest that many children with complex CHD enter the world with a brain that is already biologically vulnerable. This vulnerability may heighten their sensitivity to environmental experience across development, such that exposure to adversity may exert stronger ND effects than it otherwise might in typically developing populations. For example, within the context of complex CHD, white matter dysmaturation may amplify deprivation effects via reduced network integration, while altered limbic-prefrontal circuitry may increase threat sensitivity. Specific hypotheses of this sort would be plausible within a dimensional model.

### CHD environments and exposures

**I**n addition to the inherent brain and biological vulnerabilities associated with cardiac disease, children with complex CHD are also exposed to a constellation of environmental experiences that align, in some ways, with the dimensional constructs of deprivation and threat.

There are at least seven primary and partially overlapping environments/contexts in which CHD-related medical-adversity may occur: 1) the prenatal environment, 2) the perioperative and intensive care environment, 3) the chronic medical management environment, 4) the caregiving and family environment, 5) the developmental opportunity environment, 6) the educational and social environment, and 7) the structural and socioeconomic context. The remainder of this section will highlight selective relevant exposures within these environments and how those exposures may be conceptualized according to a dimensional framework (see Table 1).

**Table 1:** CHD environments and dimensional adversity exposures.

CHD Environment	Deprivation Exposures *(Absence of developmentally appropriate inputs/opportunities)*	Threat Exposures *(Experiences involving harm, fear, or safety signals)*
Prenatal	• (Less clearly implicated) • Possible indirect effects on reduced optimal prenatal developmental conditions	• Fetal exposure to maternal distress signaling threat
Perioperative & Intensive Care	• Prolonged sedation • Restricted movement/exploration • Child-caregiver separation • Limited caregiver interaction (holding, feeding, talking) • Sensory dysregulation (over/under stimulation)	• Painful and invasive procedures • Exposure to alarms, emergencies • Use of restraints
Chronic Medical Management	• Disruption of routines • Reduced participation in school, sports, peer activities	• Repeated painful procedures and testing • Anticipatory anxiety about procedures/results • Chronic physiological threat (e.g., hypoxemia, low cardiac output)
Caregiving & Family	• Reduced caregiver availability/responsiveness • Inconsistent routines • Overprotection limiting autonomy development • Reduced sibling interaction • Less family/social time	• Exposure to caregiver distress, anxiety, trauma • Child sensitivity to caregiver threat cues • Family stress and instability • Sibling psychosocial distress contributing to stressful environment
Developmental Opportunity	• Limited physical activity • Restricted exploration (due to hospitalization, equipment) • Reduced participation in sports/extracurriculars	• Fear-based self-limitation of activities • Parental restriction due to safety concerns
Educational & Social	• Frequent school absences • Limited peer interaction (especially during hospitalizations) • Reduced participation in social activities	• Bullying, teasing, social exclusion • Perceived “difference” by peers
Structural & Socioeconomic	• Limited access to healthcare • Reduced access to ND follow-up care • Fewer developmental resources/opportunities	• Neighborhood violence • Domestic violence • Systemic racism

CHD, congenital heart disease.

#### Prenatal environment

Substantial evidence highlights the important role of parental stress and mental health during pregnancy and its impact on brain development. Wu and colleagues (59), for example, reported associations between elevated maternal distress (i.e., stress, anxiety, depression) during pregnancies complicated by a CHD diagnosis and reduced fetal hippocampal and cerebellar volumes – two brain areas that are particularly vulnerable to being disrupted by prenatal stress (60) as well as by postnatal threat (61) and deprivation (62), respectively. In a recent follow up study, this same group (63) reported longitudinal associations between elevated prenatal maternal distress and impairments in 18-month motor development, as well as higher parent-reported externalizing behavior problems. Interestingly, prenatal distress did not predict cognitive or language outcomes, suggesting perhaps that exposure to elevated prenatal stress in CHD may be better characterized along the threat than deprivation dimension of adversity.

#### Perioperative & intensive care environment

The perioperative and intensive care setting is a complex environment in which it can be exceedingly difficult to appropriately calibrate the kind and intensity of various forms of stimulation to the unique needs of the child with CHD (64–66). Prolonged postoperative sedation, restricted opportunities for movement and exploration, inappropriate sensory stimulation (e.g., auditory, visual, tactile), frequent child-caregiver separations, and reduced opportunities for parents to hold and feed and talk to their babies – all of these realities of the intensive care environment may conspire to deprive children with CHD of the kinds of species-typical stimulation and reciprocal interactions that support early brain development, as well as child-caregiver bonding and attachment (66–68).

At the same time, the perioperative and intensive care setting is also one in which many children with CHD are exposed to necessary yet stressful and painful medical/surgical procedures, the use of physical restraints during procedures, and/or exposure to nearby alarms and emergency responses. Each of these exposures may signal a threat to the child's physical health or safety and therefore have the potential to trigger their stress- and threat-response systems.

#### Chronic medical management environment

CHD is a chronic disease that requires ongoing medical management long after children leave the perioperative/intensive care setting. For some children with complex CHD, chronic hypoxemia and/or low cardiac output present persistent biological vulnerabilities that threaten both their mental and physical health and may limit their involvement in developmentally appropriate activities. But even in the absence of significant ongoing cardiac dysfunction, the need for painful outpatient procedures and frequent diagnostic testing, coupled with the anxiety that many children and adolescents with CHD experience in anticipation of upcoming procedures/testing (and while awaiting test results), may constitute recurrent threat exposures within the chronic medical management environment.

Moreover, the need to carefully monitor their physical status and attend frequent medical appointments may disrupt routines and limit opportunities to engage in other salient developmental experiences (e.g., attending school, playing sports, spending time with friends).

#### Caregiving & family environment

Parent and caregiver mental health is vital to child neurodevelopment. Among the parents of children with complex CHD, 25%–50% report significant symptoms of anxiety and/or depression; 30%–80% report severe psychological distress; and >80% endorse at least one symptom consistent with trauma (69).

Parents who are highly stressed, depressed, and/or traumatized may be less available to care for their child, and less consistent/predictable in maintaining routines and responding to their child's needs. Parents who are anxious and/or depressed may also be hypervigilant to threats in the environment (70), which may contribute, in part, to the excessive limit-setting and overprotection that is commonly observed among children with CHD (71). Exposure to age-appropriate challenges fosters the development of autonomy, whereas overprotection may limit a child's opportunity to overcome challenges and master their environment.

Children are acutely sensitive to detecting threat cues from their primary caregivers and others around them, through social referencing. When parents are struggling to manage their own fears and/or trauma, children may interpret that as cause to be fearful themselves.

CHD can also disrupt the broader family environment in ways that impact child ND and psychosocial outcomes. When a child with CHD and their parent need to be away for medical appointments or hospitalizations, they may be separated from their other caregivers, siblings, and/or extended family members who remain at home for financial, school, and other reasons. Reduced time with siblings is particularly notable given that sibling relationships are often among the earliest opportunities a child has to navigate interpersonal interactions with other children. Parents of children with CHD do indeed report concerns about having less time for their family members to be together (72) and less time for social activities (73), which may represent sources of deprivation for the child with CHD – as well as poorer psychosocial adjustment among the siblings themselves (74), which may add further threat and stress to the family environment.

#### Developmental opportunity environment

Across settings, complex CHD may undermine opportunities to engage in activities that support neurodevelopment. During infancy, prolonged hospitalization, sedation, pain, and medical equipment (e.g., lines, tubes) may restrict movement and limit opportunities to explore and master the environment.

Outside the hospital, limitations on physical activity and/or parental overprotection may prohibit some children and adolescents with CHD from engaging in preferred sports and extracurricular activities. Some children/adolescents may also self-limit out of fear that physical activity may be harmful to them.

#### Educational & social environment

Children and adolescents with CHD may experience more frequent school absences than their peers due to illness, medical appointments, and/or hospitalizations. While in the hospital setting, their ability to interact socially with peers is likely to be substantially limited, although this may be mitigated to some degree by social media.

At school and within the community, children and adolescents with CHD may be perceived by others as “different,” which may increase their risk for exclusion and/or teasing/bullying. Their availability to engage in social activities may also be limited on account of their medical condition.

#### Structural & socioeconomic context

Socioeconomic status (SES) is a powerful predictor of ND and mental health outcomes in children/adolescents with CHD (24). According to longitudinal data from the Boston Circulatory Arrest Study, SES-related deficits in neurodevelopment among children with dextro-transposition of the great arteries are detectable early, within the first year of life, with more robust and stable ND deficits apparent throughout childhood and adolescence (24). Miles et al. (75), looking at Danish population-based cohort data of individuals with CHD, found that parental socioeconomic indicators predicted several ND and mental health conditions, including anxiety/mood disorders, ADHD, and developmental disability. Moreover, children with CHD who are from lower SES backgrounds are more likely to have been diagnosed with CHD postnatally (25, 76), which carries an increased risk for perioperative brain injury (77) and poorer ND outcomes (78). They are also less likely to attend cardiac ND follow up clinic (79), further limiting their access to recommended care and monitoring (1, 80, 81).

Socioeconomic disadvantage often reflects a combination of adverse exposures, including deprivation (e.g., lack of developmentally appropriate stimulation, limited access to healthcare) and threat (e.g., neighborhood violence, domestic violence, systemic racism). While often correlated, these exposures can be empirically and phenomenologically disentangled (26, 32, 82), for example, in the case of a family experiencing financial strain due to medical costs that nonetheless lives in a safe neighborhood within a supportive and close-knit community.

## A dimensional model of neurodevelopment in CHD

**Figure 1:**
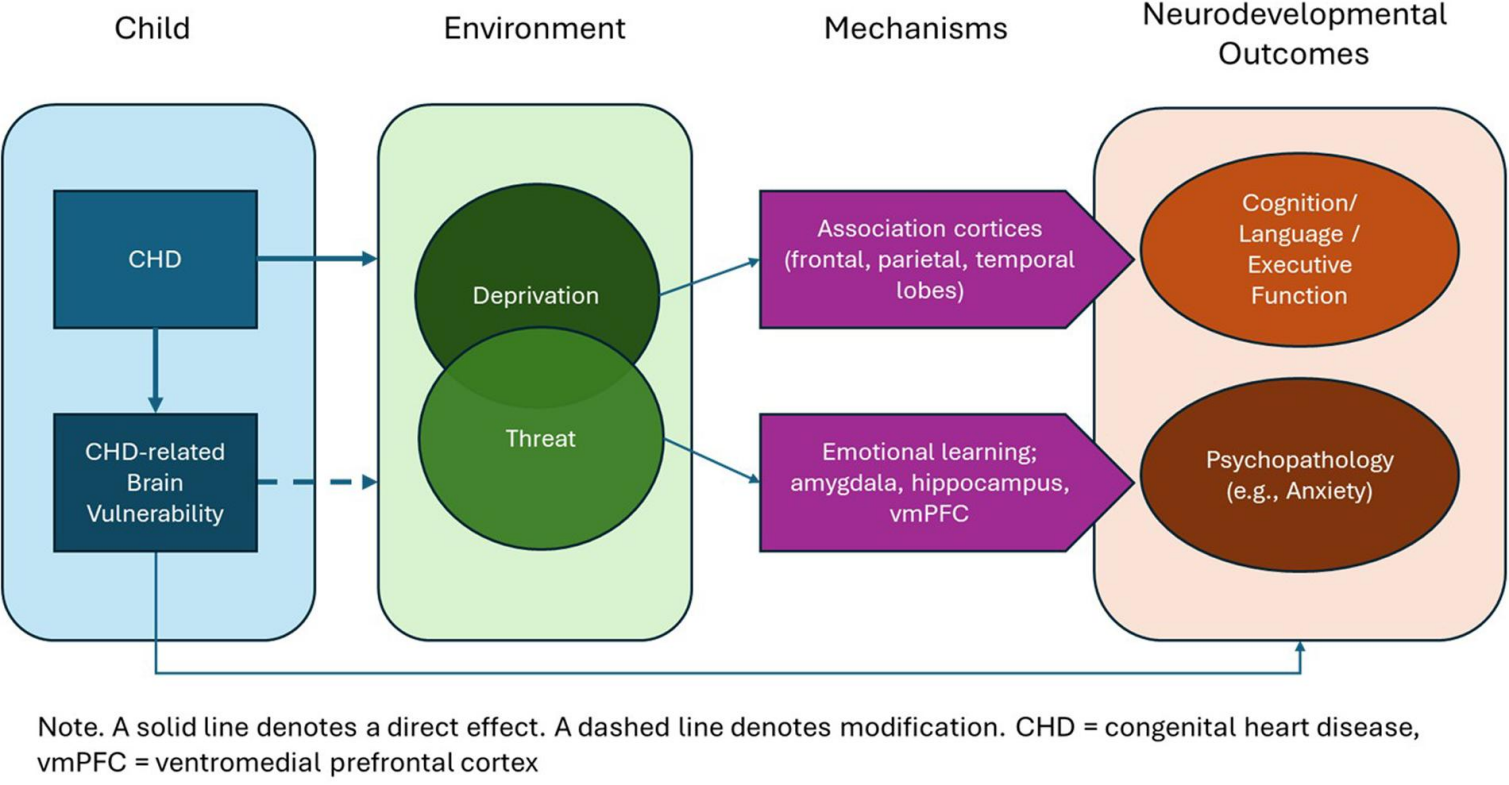
A dimensional model of neurodevelopment in congenital heart disease.

The proposed model of ND outcomes in children with CHD reflects the direct and interacting contributions of CHD-related brain vulnerability and exposure to environmental medical adversity (Figure 1).

CHD-related biological vulnerability includes alterations in brain structure and function arising from prenatal dysmaturation, perioperative injury, and cardiac disease-related physiological factors. These alterations are not entirely deterministic of ND outcomes, as evidenced by significant variability in outcomes among individuals with CHD, including those with similar cardiac anatomy and/or neuroimaging findings. Nor do they act solely as modifiers of environmental risk. Rather, CHD-related brain alterations are hypothesized to impact neurodevelopment both independently and conditionally, via their modification of environmental sensitivity to adversity.

Environmental adversity exposures are organized along two dimensions: deprivation and threat. These dimensions frequently co-exist but are not entirely colinear and therefore can be analytically separated.

Deprivation and threat are hypothesized to influence neurodevelopment among children with CHD through partially distinct neurobehavioral pathways. Deprivation, through its impact on distributed association cortices throughout the brain, is predicted to preferentially undermine cognition, executive function, and language development. Threat, via its impact on emotional learning and stress-regulation systems, is predicted to increase risk for emotional dysregulation and psychopathology, particularly anxiety and mood disorders.

In children with complex CHD, medical-adversity exposures (e.g., hospitalization, painful procedures, caregiver separations) can be characterized along these two dimensions (i.e., deprivation and threat). The ND impact of these exposures is not absolute, however, and likely depends on CHD-related brain vulnerability, which may independently and interactively determine each child's unique sensitivity to environmental input.

## Discussion

### Theoretical context

The proposed model is grounded in existing developmental theory. In structural terms, Bronfenbrenner's *ecological systems theory* (83), Sameroff's *transactional model of development* (84), and several core tenets of *developmental psychopathology* as formulated by Cicchetti (85), Sroufe and Rutter (86), and others, provide the foundation upon which an understanding can be built of *where* development occurs, *how* children and environments influence one another, and *why* developmental pathways diverge. Building upon these perspectives, McLaughlin and Sheridan's *DMAP*, as discussed above, provides a more mechanistic account of *which* aspects of environmental experience are likely to influence particular neurodevelopmental systems.

Bronfenbrenner's (83) ecological systems theory (which later evolved into the bioecological model (87) holds that development occurs within the context of multiple nested environments. In the case of cardiac ND research, the field has increasingly recognized the importance of various environmental contexts on child outcomes. In identifying seven environmental contexts thought to be particularly relevant to the lived experiences of children with CHD – and highlighting developmentally meaningful manifestations of deprivation and threat *within* those environments – the proposed model is consistent with ecological systems theory while offering a domain-specific and mechanistically informative account of the kinds of experiences within those environments that are likely to influence neurodevelopment.

Looking more specifically at the processes through which children and their various environments interact, Sameroff's (84, 88) transactional model of development posits that development results from the bidirectional influences of child and context. Among its many contributions to the field was the observation that a child's temperament may actively elicit different patterns of responsivity from their parents – which, in the case of some particularly challenging temperamental styles, may contribute to a mismatch between the care a child most needs and the care they are most likely to receive. While the proposed dimensional ND framework does not explicitly model these bidirectional processes, it is nonetheless compatible with transactional theory, particularly in its deliberate inclusion of family/parental mental health and overprotection as salient influences on neurodevelopment in the context of complex CHD.

Finally, the proposed dimensional model shares many core assumptions with developmental psychopathology, including that development is probabilistic rather than deterministic; that an understanding of atypical development requires an understanding of typical development, and vice versa; that different developmental pathways can converge on a common outcome (i.e., equifinality); that the timing of an exposure is key to understanding its potential impact on development; that a common developmental starting point can lead to different outcomes for different children (i.e., multifinality); and that development is complex and multiply determined and therefore must be examined via multiple levels of analysis (e.g., genetic, neural, psychological, social (85, 86, 89, 90);. These principles are fully consistent with the proposed dimensional model. Indeed, notions of developmental heterogeneity and multifinality are particularly germane considering known variability in ND outcomes among individuals with CHD despite seemingly similar diagnoses and treatment courses.

In summary, the proposed dimensional model of cardiac ND builds upon well-established developmental theory. Rather than offering an alternative philosophy of development or refutation of existing theory, the proposed model seeks to operationalize medical experiences in developmental terms by characterizing them along the dimensions of deprivation and threat, thereby providing a more mechanistic account of how those experiences may influence development in the context of complex CHD.

### Advantages of a dimensional model of neurodevelopment in CHD

A dimensional model of neurodevelopment in CHD offers at least four potential advantages for research and clinical practice. First, it provides a more mechanistic framework for understanding ND outcomes in CHD. Rather than treating risk factors as independent predictors, the model conceptualizes neurodevelopment as the probabilistic result of children's exposure to environmental adversity occurring within the context of varying degrees of underlying brain vulnerability. In this framework, brain vulnerability may amplify the developmental impact of deprivation and threat exposures. This allows the model to more closely reflect the lived experiences of children with CHD across multiple developmental contexts.

Second, by incorporating neuroscientific evidence regarding the differential sensitivity of specific brain systems to distinct dimensions of adversity, this model enables more precise predictions regarding ND outcomes. See Table 2 for five testable hypotheses engendered by a dimensional model of ND in CHD. Researchers may be able to begin testing some of these hypotheses using existing data, re-coded to more accurately reflect deprivation and threat dimensions; others will require the collection of new data.

**Table 2:** Five testable hypotheses generated by a dimensional model of cardiac neurodevelopment.

#	Hypothesis
1	CHD-related deprivation exposures (e.g., caregiver separation, prolonged sedation) will be more strongly associated with cognitive deficits than CHD-related threat exposures.
2	CHD-related threat exposures (e.g., invasive medical procedures, family stress and instability) will be more strongly associated with emotion dysregulation/psychopathology than CHD-related deprivation exposures.
3	When modeled simultaneously, deprivation-related exposures will be uniquely predictive of cognitive (but not psychopathology) outcomes, while threat-related exposures will be uniquely predictive of psychopathology (but not cognitive) outcomes.
4	The association between CHD-related deprivation exposures and cognitive outcomes will be stronger for children with greater prevalence/severity of CHD-related brain vulnerability (e.g., white matter injury).
5	The association between CHD-related threat exposures and psychopathology will be stronger for children with greater prevalence/severity of CHD-related brain vulnerability (e.g., altered limbic-prefrontal circuitry).

CHD, congenital heart disease.

Third, improved mechanistic understanding and greater predictive specificity have the potential to inform more effective prevention and intervention strategies. By identifying the developmental pathways through which specific exposures influence specific neurobehavioral outcomes, a dimensional framework could guide both broad preventive efforts and targeted interventions aimed at reducing deprivation and mitigating threat exposure in the lives of children with CHD and their families. For example, within the intensive care unit, efforts to reduce deprivation-related experiences may focus on reducing caregiver separations, supporting language exposure, and implementing mobility protocols, while efforts to reduce threat-related exposures may prioritize preparation for painful procedures and trauma-informed care practices.

Finally, the adoption of a dimensional model for cardiac ND research may also open new avenues of study that have been overlooked to date. “Hidden talents” research, for example, represents an exciting new direction in developmental science that emerged from the realization that we know a lot more about the ways adversity can undermine development, but fairly little about the adaptive skills and traits that may develop in response to stress and adversity (91, 92). Studies aimed at understanding the kinds of “stress-adapted skills” (91) that help children with CHD to survive within the context of CHD-related deprivation and threat (but perhaps become less adaptive in more optimal environments) would represent a new frontier for cardiac ND research, and would perhaps bring a sense of balance and positivity to a field that has to date been primarily deficit-focused.

### Challenges and future directions

The proposed model, though grounded in well-established developmental theory and solid empirical evidence, nonetheless faces several potential challenges. Some are conceptual/definitional, such as the appropriateness of describing CHD-related experiences as sources of “adversity.” Others are more pragmatic in nature, having to do with measurement and our ability to parse CHD-related exposures dimensionally. Still others highlight acknowledged uncertainties and gaps that may be filled through future theorizing and investigation, ostensibly leading to further refinement of the model. A discussion of these and other challenges and future directions follows below.

#### Can medical experiences really be considered “adversity”?

While much dimensional adversity research comes from studies of child maltreatment (e.g., abuse, neglect) or institutional rearing, the proposed model does not claim that CHD-related experiences are equivalent to maltreatment or institutionalization, only that they share certain developmentally salient qualities with these more extreme environmental conditions. Unlike child maltreatment, which is typically perpetrated by a parent or family member, it is the CHD/medical experience itself that may limit a child's opportunities for developmentally appropriate stimulation (i.e., deprivation) and/or expose a child to painful, scary situations (i.e., threat).

Moreover, whereas child abuse by a caregiver creates an unresolvable paradox wherein the caregiver's role is simultaneously that of support system and victimizer (93), in CHD the parent/caregiver is not directly causing harm (although, in some instances, they may be perceived by the child as failing to prevent it) and, ideally, is able and sufficiently empowered to serve as a critical buffer between the child and their stressful circumstances. So, while there are undeniably substantive differences between maltreatment/institutionalization and CHD, there are also underlying, often less extreme commonalities that should be explored.

#### Do dimensional models of adversity only pertain to maltreatment or institutionalization?

A somewhat different but related question is whether dimensional adversity models such as DMAP pertain only to the most extreme forms of adversity, such as maltreatment and institutionalization. In fact, there is substantial evidence attesting to the broader population-level relevance of the DMAP. Using data from nearly 12,000 children and adolescents who participated in the Adolescent Brain Cognitive Development (ABCD) study, for example, Russell and colleagues (94) looked at a broad range of “traumatic and adverse childhood experiences” (TRACES), including family conflict, caregiver maladjustment, and poverty. At baseline, nearly all of these TRACES correlated with poorer psychosocial adjustment and cognitive ability; however, over time – and consistent with dimensional models – exposures reflecting deprivation (e.g., poverty, caregiver maladjustment) were more strongly predictive of declines in cognitive status while those capturing threat (e.g., family conflict) were more strongly predictive of increases in psychopathology. Further supporting the generalizability of dimensional models are studies examining cognitively stimulating home environments, including among children living in low- or middle-income settings (95) and also children with CHD (96), which consistently demonstrate links between environmental stimulation/deprivation and child cognitive development. Taken together, there is sufficient evidence, therefore, to conclude that deprivation and threat are not only relevant in their most extreme forms but also carry the potential to impact development in less severe forms, including those forms experienced by children with complex CHD.

#### How can deprivation and threat exposures be operationalized within the intensive care setting?

Both deprivation-related and threat-related exposures coexist within the perioperative and intensive care environment, and these dimensions are likely to be correlated. For example, during a single prolonged intensive care admission, a child may endure reduced opportunities for expectable developmental experiences (i.e., deprivation) and exposure to painful procedures (i.e., threat). This does not mean, however, that these experiences cannot be measured and analyzed with respect to their respective impacts on child neurodevelopment.

For example, a prospective longitudinal study could recruit consecutive intensive care admissions of infants undergoing surgery with cardiopulmonary bypass and systematically quantify deprivation and threat exposures throughout the perioperative hospital stay. Deprivation-related indices could include amount and quality of parent-infant interaction, quantity and quality of sensory exposures (e.g., sound, light), and duration of restricted movement/exploration due to sedation, while threat-related indices could include cumulative exposure to painful procedures, number/duration of invasive lines and tubes, and emergent medical events. Neurodevelopment (via standardized testing) and neuroanatomical outcomes (via neuroimaging) could subsequently be examined in relation to these exposures using analytic approaches that account for their correlation and estimate unique contributions, while controlling for pertinent confounders (e.g., genetic status). This approach would also provide a framework for evaluating whether intensive care-based interventions such as individualized developmental care (66, 67) reduce deprivation and/or threat exposures and whether such reductions are associated with improved ND outcomes.

#### What about unpredictability?

Dimensional models of adversity have come increasingly to recognize *unpredictability* as yet another salient contributor to development and psychopathology. Defined broadly as variability in environmental conditions/harshness over time (26), research has linked early unpredictability (operationalized variously as family instability, inconsistent caregiving, inconsistent parental mental health, inconsistent parenting behaviors, residential unpredictability, financial unpredictability, etc.) to a wide range of cognitive, emotional, and behavioral outcomes (97–100).

Unpredictability and uncertainty are often identified as sources of stress for the parents of children with CHD (3). Even after surgery, parents endorse feeling as though they are “not out of the woods” because their child's status could potentially change at any time, without warning (101). Children and adolescents with CHD likewise identify “enduring uncertainty and medical adversity” as a core theme of their lived experience of CHD (102).

It seems, therefore, that unpredictability is indeed germane to any discussion of “what having CHD is like” for children and families. Nonetheless, there are at least two reasons for not including unpredictability within the proposed dimensional model of cardiac neurodevelopment.

Firstly, despite having clear significance for development, unpredictability does not predict development or psychopathology outcomes with the same degree of specificity as deprivation or threat. Some of this lack of specificity may stem from the multitude of ways unpredictability has been conceptualized and defined across studies. Operationalizing unpredictability within the context of CHD would likely also be quite challenging, in part because the kinds of CHD-related unpredictability/uncertainty described by parents and children seem to coexist with – and may be difficult to disentangle from – deprivation/threat (e.g., a sudden decompensation is both threatening and unpredictable; the unanticipated need for surgery may follow after months of anticipatory worry).

Second, while there is evidence that unpredictability, deprivation, and threat are distinct dimensions of adversity (26, 82), there are also models wherein unpredictability is cast in a modifying role, modulating the impact of deprivation and threat on subsequent outcomes, at times with disparate or even paradoxical effects. Farkas and Jacquet (103), for example, found that while deprivation and threat were associated with decreased future orientation (i.e., one's inclination to think ahead and plan for the future) and psychological distress, respectively, unpredictability of deprivation/threat seemed to function as a partial buffer against those same negative outcomes.

These challenges notwithstanding, future work should further explore unpredictability in the context of CHD, including its potential integration within the proposed dimensional model. One particularly interesting future direction would involve the use of ecological momentary assessment (EMA) to capture real-time, moment-to-moment parent-child interactions within the cardiac intensive care setting, to examine fluctuations in parental stress/mood and their impact on child neurodevelopment. MacNeill et al. (104) employed a similar approach among 72 pregnant persons (in pregnancies not affected by a prenatal CHD diagnosis), and found that parental stress lability during pregnancy predicted higher infant negative affectivity at 3 months. This would be worth exploring in pregnancies complicated by a CHD diagnosis, including as a potential outcome of interest in prenatal psychosocial intervention studies [e.g., HEARTPrep (105), Heart GPS (106)].

## Conclusion

Cardiac ND research has progressed tremendously over the past 40 + years. However, despite major advancements in describing outcomes and identifying risk factors, ND status in children with CHD has shown little improvement over time, about 70% of outcome variability is unexplained, and clinical prediction of individual prognosis remains poor. Overcoming these major obstacles will be necessary if we are to truly achieve a future in which optimal neurodevelopment is the reality for every one of the >13 million (107) individuals with CHD worldwide.

Throughout this article, I have laid out a mechanistic developmental model wherein neurodevelopment among children with CHD is conceptualized as the probabilistic outcome of the independent and interactive effects of CHD-related neurological vulnerability and exposure to environmental medical adversity, as characterized by dimensions of deprivation and threat – each of which is posited to differentially impact specific ND systems. Grounded in developmental science, this novel synthesis of theory and evidence highlights the critical role of the medical system in creating an ecology of environmental experiences that restricts access to developmentally appropriate stimulation (i.e., deprivation) and compromises safety and security (i.e., threat).

This model invites a shift from cataloging risks to identifying how specific CHD-related environmental exposures shape distinct neurobehavioral outcomes. In so doing, it provides a framework for moving beyond risk identification toward targeted intervention. Although important conceptual and logistical challenges remain, this approach has the potential to inform more precise and developmentally grounded clinical care across the multiple environments in which children with CHD grow and develop. Its ultimate value will depend on whether it leads to improved prediction and, critically, to better ND outcomes for children with CHD.

## Data Availability

The original contributions presented in the study are included in the article/Supplementary Material, further inquiries can be directed to the corresponding author.
